# Epidemiologic surveillance of nosocomial infections in a Pediatric Intensive Care Unit of a developing country

**DOI:** 10.1186/1471-2431-10-66

**Published:** 2010-09-10

**Authors:** María R Becerra, José A Tantaleán, Víctor J Suárez, Margarita C Alvarado, Jorge L Candela, Flor C Urcia

**Affiliations:** 1 Pediatric Intensivist, Instituto Nacional de Salud del Niño, Lima, Peru; 2 Pediatric Intensivist, Master in Medicine, Instituto Nacional de Salud del Niño and Universidad Nacional Federico Villarreal, Lima, Peru; 3 Infectologist, Tropical Infectious Diseases, Instituto Nacional de Salud, and Universidad César Vallejo, Lima, Peru; 4 Nurse, Instituto Nacional de Salud del Niño, Lima, Peru; 5 Pediatric Infectologist, Instituto Nacional de Salud del Niño, and Universidad San Martín de Porres, Lima, Peru; 6 Biologist, Instituto Nacional de Salud, Lima, Peru

## Abstract

**Background:**

Nosocomial Infections (NI) are a frequent and relevant problem. The purpose of this study was to determine the epidemiology of the three most common NI in a Pediatric Intensive Care Unit from a developing country.

**Methods:**

We performed a prospective study in a single Pediatric Intensive Care Unit during 12 months. Children were assessed for 3 NI: bloodstream infections (BSI), ventilator-associated pneumonia (VAP) and urinary tract infections (UTI), according to Center for Disease Control criteria. Use of devices (endotracheal tube [ETT], central venous catheter [CVC] and urinary catheter [UC]) was recorded.

**Results:**

Four hundred fourteen patients were admitted; 81 patients (19.5%) developed 85 NIs. Density of incidence of BSI, VAP and UTI was 18.1, 7.9 and 5.1/1000 days of use of CVC, ETT and UC respectively. BSI was more common in children with CVCs than in those without CVCs (20% *vs*. 4.7%, p < 0.05). *Candida *spp. was the commonest microorganism in BSI (41%), followed by Coagulase-negative *Staphylococcus *(17%). *Pseudomonas *(52%) was the most common germ for VAP and *Candida *(71%) for UTI. The presence of NI was associated with increased mortality (38.2% *vs*. 20.4% in children without NI; p < 0.001) and the median length of ICU stay (23 *vs*. 6 days in children without NI; p < 0.001). Children with NI had longer average hospital stay previous to diagnosis of this condition (12.3 *vs*. 6 days; p < 0.001).

**Conclusions:**

One of every 5 children acquires an NI in the PICU. Its presence was associated with increased mortality and length of stay. At the same time a longer stay was associated with an increased risk of developing NI.

## Background

Nosocomial Infections (NIs) are a frequent problem, particularly in Intensive Care Units (ICU). In Europe, incidences range from 1% in General Pediatric wards up to 23.6% in Pediatric ICUs (PICU) [1]. PICU studies report incidences between 6.1 - 15.1% [2,3] whilst a cross-sectional study found a prevalence of 11.9% [4].

Bloodstream infections (BSI) are the most common NI in PICUs (28-52% of all) [2-6]; pneumonia (including ventilator associated pneumonia, VAP) and urinary tract infection (UTI), followed by enteric, surgical site and skin [1-4]. BSI and pneumonia are responsible for approximately 50% of NIs, and UTI causes an additional 12-22% [3,7]. Although VAP is the most frequent NI in adults (8 - 28% of adults under mechanical ventilation, MV, developed VAP [8]), pediatric studies report incidences of 2 - 17% [9-12]. Only two pediatric studies have found VAP to be the most frequent NI in PICU [1,13].

All aforementioned studies, except two [12,13], come from developed countries. In contrast to PICUs from developed countries, those in developing countries often admit more critically ill patients, with medical conditions rather than surgical, and with lower ages and socioeconomic level [14]. A study from Brazil found that yeasts and Gram negative bacteria were the most frequent isolates in blood cultures [13]. Another study in adults from developing countries found greater frequency of NI compared to ICUs in the USA, despite similar device use rates [15]. So, it is possible that there could be differences in the demographic and epidemiologic characteristics of NI in PICUs from developing countries. The objective of this study was to understand the epidemiological profile of the 3 most common NIs (BSI, VAP and UTI) in a PICU of a developing country.

## Methods

A prospective study, which followed rules of active epidemiologic surveillance, was performed in the PICU at the Instituto Nacional de Salud del Niño (INSN) of Lima, Peru. This is a national reference center admitting patients from all over the country. It has 16 beds for children between 0 and 18 years, admitting around 500 patients per year. Cardiac surgery and burn patients have a dedicated ICU. Usually no preterm infants are admitted to the PICU, and no prophylactic antibiotic or antifungal therapy is given for high risk newborns. Most admissions come from the Emergency Department.

This study, approved by the Committees on Ethics of the INSN and the Instituto Nacional de Salud (INS) of Lima occurred over a twelve month period (June 21st 2006 through to June 20th 2007).

The CDC diagnostic criteria [16] were used, along with those of the International Pediatric Sepsis Consensus Conference [17], as was recently proposed [18]. An infection was defined as nosocomial when it appeared after 48 hours of PICU admission and if there was no evidence that the infection was present or incubating at the time of PICU admission.

Diagnosis of BSI included both laboratory confirmed infections, and clinical sepsis. Clinical sepsis was defined as such when a physician prescribed treatment for sepsis, in a patient with signs and symptoms of sepsis with no other apparent site of infection, and blood culture was negative or no blood culture was performed. Laboratory confirmed infection was diagnosed when: a) a recognized pathogen was isolated from one or more blood cultures and this pathogen was not associated to infection at another site in a patient with signs or symptoms of severe infection *or*; b) a patient had a diagnosis of sepsis *and *a common skin contaminant (like coagulase-negative *Staphylococcus*, CNS) was isolated from two or more blood cultures drawn on separate occasions, *or *when it was isolated in a patient with an intravascular line *and *had treatment for sepsis prescribed by a physician. BSI was diagnosed in both, children with and without CVC. Blood samples were obtained from a separate peripheral venipuncture after skin preparation with iodine solution, in amounts according to patient's age (< 1y: 1 - 2 ml; 1 - 12 y: 1 - 10 ml; > 12 y: 10 ml).

VAP was defined as early when it developed during the first 4 days under MV, and late VAP when it developed after the fifth day [19]. Tracheobronchial samples were collected through deep tracheal aspirates from endotracheal tubes (ETT) with a closed suction technique and urine samples were aseptically aspirated from the sampling port of the urinary catheter (UC). No invasive technique for sampling of tracheal secretions was used.

UTI was classified as symptomatic UTI, asymptomatic bacteriuria and other UTIs, as defined by CDC criteria [16]. To consider urine samples as positive the number of colony forming units (CFU/ml) had to be greater than 10^5 ^CFU/ml.

All samples (blood, tracheal and urine) were obtained only after an infection was suspected. All samples were collected in containers with antibiotic remover (BACTEC, Becton Dickinson, Microbiology system, Cockeysville, MD, USA) and processed in the INSN Laboratory. Some isolated bacteria were sent to the INS to confirm bacterial susceptibility by disk diffusion method. All yeasts isolated at the INSN were sent to the INS and antifungal susceptibility was determined by disk diffusion method.

Other NIs such as upper respiratory, surgical, enteric, skin or infections at other sites, were not surveyed. All admitted children were assessed daily (Monday to Saturday) by one of the authors (MRB, MCA or JAT) who ascertained the presence of signs compatible with NI and use of devices (CVC, ETT or UC) up to 48 hours of discharge from the PICU.

At the time of the study there were no approved rules for insertion of CVCs, but for all insertions masks, sterile gowns and gloves were used, cleaning the skin with clorhexidine 2% plus disinfection with alcohol 70% and covering the puncture site with sterile gauze and transparent dressing. Written rules for prevention of catheter-associated BSI approved by the Infection Control Committee were in use since 2003. The connecting tubes were changed every third day, unless CVCs were used for TPN or blood transfusions, in those cases it was changed every day. Any drug administration was preceded by cleansing of the three-way stopcock with 70% alcohol. The puncture site was cleansed with alcohol only if soiling was observed. There were written guides for ETT and UC care. Aspiration of secretions was made according to patient's condition, with aseptic technique, previously irrigating with sterile normal saline. All patients with UC in place had a closed drainage system, except infants under 1 month of age. Manipulation of UC was always with aseptic technique.

The total of patient-days and days of use of devices (CVC, ETT and UC) were recorded. NIs can be described by the *incidence rate *(per 100) using the number of each assessed infection as numerator and the number of patients with risk factors as denominator, or by *device-associated incidence rate *(DI) (per 1000) dividing the number of each assessed infection by the number of days which patients were exposed to risk factors. For calculating density of incidence of BSI and UTI only children with CVC or UC were considered. A ventilator, CVC or UC day was considered as such only if the device was placed during the morning hours (until 13:00). The ratio of use of devices was estimated dividing number of days of use of a device between days the patients spent at the ICU.

Crude excess mortality was defined as the difference between mortality in children with and without NI. PRISM score was calculated during the first 24 hours of admission.

We performed descriptive analysis of central tendency and variability measurements as well as rate estimates. To perform bivariate and multivariate analysis to determine the risk for NI linear generalized models with Poisson family and log link adjusted values for age, sex and PO condition were used. Furthermore, the Kaplan-Meier analysis for survival was performed for the time of the NI event at the ICU and for death at ICU. Categorical variables were evaluated using the chi square or Fisher' exact test, as appropriate. Continuous variables were evaluated using the Mann-Whitney test. Proportions were compared using the z test, median test to compare medians and Mann Whitney's U test to compare averages. We used the stata 8.0 statistical program (Single user Stata for Windows perpetual license. Serial number: 8199049125. Licensed to: Instituto Nacional de Salud).

## Results

### Demographic features and use of devices

There were 444 admissions for 414 patients (18 patients were admitted twice and 6 were admitted 3 times). The average age was 52.8 months (range: 0.02 - 214; SD 62.9; first quartile: 4.0, third quartile: 103.7) and the median was 19 months; 189 children (46%) were under 1 year. Demographic features, reason for admission, comorbidities, patient-days, use of devices, length of stay and mortality for patients with and without NI are described in Table 1. The group of children with NI was younger; however reasons for admission to PICU as well as comorbidities were similar in both groups. No preterm infant was admitted during the study period.

**Table 1:** Demographic features, comorbidities and device use in children

	WITH NI (n = 81)	WITHOUT NI (n = 363)	P	ALL (n = 444)
***AGE***				

*months, median*	7.7	24	0.002	19

***GENDER***				

*M/F*	50/31	192/171	-	242/202

***Medical n (%)***	**68 (84)**	**272 (75)**	0.053	**340 (76.6)**

*respiratory*	21	60	-	81

*neurological*	8	69	-	77

*septic*	21	49	-	70

*others*	18	94	-	112

***Post operative n (%)***	**13 (16)**	**91 (25)**	0.053	**104 (23.4)**

*neurosurgery*	2	45	-	47

*others*	11	46	-	57

***COMORBIDITY***				

**total (%)**	**45 (56)**	**163 (45)**	0.081	**208 (47)**

*congenital syndromes*	30	79	-	109

*chronic conditions*	11	52	-	63

*neoplasia*	2	26	-	28

*others*	2	6	-	8

PRISM * *average*	7 ± 6.7	6.47 ± 6.63	0.74	

**PATIENT DAYS**	2689	1914	-	4603

***DEVICE USE***				

*CVC days*	1180	922	-	2102

*ventilator days*	2091	1329	-	3420

*UC days*	620	550	-	1170

***DEVICE UTILIZATION RATIO***				

*CVC*	0.44	0.48	-	0.46

*ETT*	0.78	0.69	-	0.74

*UC*	0.23	0.28	-	0.25

***ICU STAY ****(days)median*	23	6	< 0.001	6.9

***MORTALITY***				

*PICU, n (%)*	31 (38.3)	74 (20.4)	< 0.001	105 (23.6)

*Hospital, 28 d (%)*	36 (44.4)	119 (32.7)	0.060	155 (34.9)

* data from 97 patients (20 with and 77 without NI)

NI: nosocomial infection; CVC: central venous catheter; ETT: endotracheal tube; UC: urinary catheter

### Table 1 Demographic features, comorbidities and device use in children

**Figure 1:**
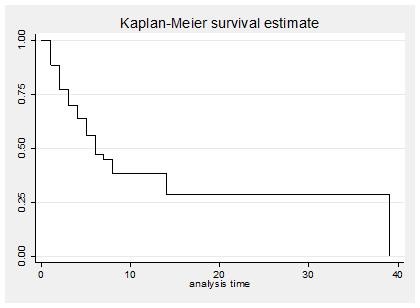
**Time of death**. The Kaplan-Meier curve describes (in weeks) the time of mortality.

Of all admissions, 52% came from the Emergency Department. Of admitted children, 43% had a CVC, 70% ETT and 38% UC. The ratios of use of devices were 0.46, 0.74 and 0.25 for CVC, ETT and UC respectively. PICU mortality for the entire population during the study period was 23.6%. The Kaplan-Meier analysis shows that 50% of deaths occurred by the sixth week at PICU (Figure 1).

### Figure 1. Time of death

#### Features of Nosocomial Infections (Table 2)

**Table 2:** Features of nosocomial infections

Infections acquired at ICU	Rate
Rate of infection (UCI)/1000	26,3

Ventilator Associated Pneumonia rate/1000	7,9

Catheter Related Blood Stream Infection rate/100	18,1

Catheter Related Urinary Infection rate/100	5,1

Calculated device-associated infection rate/1000	12,5

Out of 414 admitted patients 81 (19.5%) developed an NI, most of them in children < 1 year (56.5% of NIs) and in males (50/31). The most common NI was BSI (49 cases), 29 of them with laboratory confirmation and 20 with clinical sepsis; 38/49 BSIs presented in children with CVC; amongst these 38 catheter-related BSI some microorganism was isolated in 29, and 9 were clinical sepsis. VAP was observed in 27 cases (5 early; 22 late VAP) and symptomatic UTI in 9; 6/9 UTIs presented in children with UC. DI for BSI, VAP and UTI was 18.1, 7.9 and 5.1 per 1000 days of use of CVC, MV and UC respectively. Incidence rate for NI in children with CVC, ETT or UC was 3.0, 2.7 and 2.7 per 100 patient days, respectively. Median ICU stay before NI diagnosis was 7 days.

### Table 2. Features of nosocomial infections

Rates of incidence were: 38/190 (20%) for BSI; 27/313 (8.6%) for VAP and 6/168 (3.6%) for UTI. Rate of incidence of BSI in patients with CVC was greater than in patients without CVC (20% vs. 4.7%, p < 0.05), whilst rate of incidence of UTI in patients with UC was not significantly greater than in patients without UC (3.6% vs. 1.1%, p = 0.14).

The median length of stay (LOS) in ICU for children without NI was 6 days (range 2-60), whilst in those with NI it was 23 days (range 5-268) (p < 0.001). The LOS, previous to diagnosis of NI, in children with NI was significantly greater than LOS in children without NI (12.3 vs. 6 days, p < 0.001). The average additional stay after diagnosis of NI was 20.8 days, which accounted for 60% of stay for infected patients. Median LOS for individual NI was 22.5, 25 and 34 days for BSI, VAP and UTI, respectively.

PRISM was calculated in only 22% of patients and it was no different between those patients with NI (7) compared to those without NI (6.6) (p = 0.74).

Mortality in PICU was greater amongst patients with NI (38.3%) than in patients without NI (20.4%) (p < 0.001), with a crude excess mortality of 17.8%. Mortality after 28 days of discharge from PICU was greater in children with NI, although without statistical significance (44.4% *vs*. 32.7%; p = 0.06). Mortality for BSI, VAP and UTI was 30.6%, 51.8% and 22.2% respectively. Mortality in children with CVC and BSI was greater than in children with CVC but no BSI, with no statistical significance (42.1% *vs*. 28.9%, p = 0.135). Mortality in patients with VAP was greater than in patients without VAP (51.8% *vs*. 31.1%, p < 0.05). Mortality in patients with VAP was 59% (late) and 20% (early). Mortality for UTI (22.2%) was not different from that of the entire population (23.6%).

### Distribution of pathogens

#### BSI (29 isolates)

**Figure 2:**
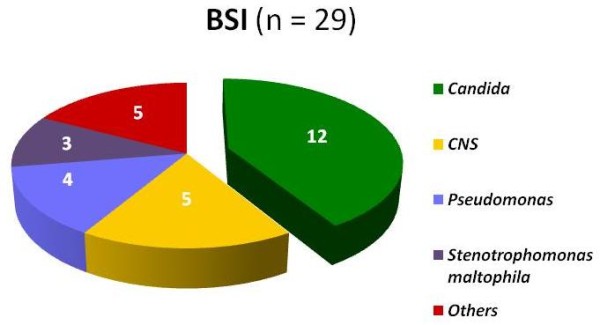
**Pathogens isolated in ICU patients with BSI**. *Candida spp *was the most common microorganism in bloodstream infection.

In 59% of cases of BSI at least one microorganism was isolated in blood cultures. The most common was *Candida *(41% of isolations) of which 10 (83%) were *non-albicans (*two cases of *glabrata, krusei, tropicalis *each; one case of *lipolitica, guilliermodi, parapsilosis*, sp each) and 2 (17%) were *C albicans. Candida *was only isolated from children with CVC. *Enterococcus *was not isolated. Figure 2 shows pathogens isolated in BSI. Out of 4 isolated *Pseudomonas*, 3 were *aeruginosa *and one *fluorescens; *included in "others" were the following: *Klebsiella oxytoca, Acinetobacter sp, Providencia stuartii, Acremonium sp *and *Kodamaea ohmeris *(one each).

### Figure 2. Pathogens isolated in ICU patients with BSI

#### VAP (31 isolates)

**Figure 3:**
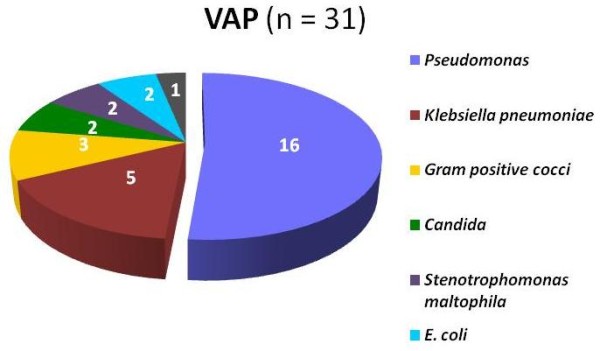
**Pathogens isolated in ICU patients with VAP**. Gram negative predominated in VAP. Only one *S aureus *was isolated.

In 97% of VAPs at least one microorganism was isolated from tracheal aspirate, *Pseudomonas *(52%) was the most common isolate. In 6 children, more than 1 microorganism was isolated. Gram negatives were less common in early VAP. Eleven multiresistant *Pseudomonas *were detected, 7 of them were isolated during the first 3 months of the study. This lead to introduce effective control measurements for NI. Figure 3 shows pathogens isolated in VAP. Out of 16 *Pseudomonas*, 15 were *aeruginosa *and 1 *fluorescens*; isolated Gram positive cocci were *S aureus, S cohnni y Streptococcus mitis *(one each); one *C albicans *and one *C glabrata *were isolated.

### Figure 3. Pathogens isolated in ICU patients with VAP

#### UTI (7 isolates)

In 86% of cases at least one microorganism was isolated. *Candida *was isolated in 5/7 cases (4 *albicans *and 1 sp); One *Klebsiella pneumoniae *and one *E coli *were isolated.

All strains (5) of CNS causing BSI, were resistant to oxacillin and susceptible to vancomycin. All isolated fungi strains (except *C krusei*) were sensible to fluconazole and voriconazole. *Pseudomonas *isolated in ETA had high antibiotic resistance rates: 13/16 for ceftazidime, 14/16 for amikacin and ciprofloxacin and 12/16 for meropenem.

Six strains produced extended-spectrum-beta-lactamase (ESBL): one of 3 isolations of *E. coli *and 5 of 10 isolations of *Klebsiella*. Four strains were isolated from ETA (one *E. coli *and 3 *K. pneumoniae*); two others came from blood cultures (*K. oxytoca*) and urine culture (*K. pneumoniae*). No ESBL producing strain was resistant to meropenem.

The bivariate analysis adjusted by sex, age and postoperative status found that the use of ETT (RR 22.9; CI 5.6,93.8), CVC (RR 3.6; CI 2.2,5.9) and UC (RR 2.0; CI 1.2,3.1) increased the risk of developing an NI. Using two devices increased the risk for NI (RR 2.6; CI 1.3,4.9), as well as using three devices (RR 3.6; CI 1.9,7.1). A 45 day stay doubled the risk for NI (RR 1.95; CI 1.6,2.3). Use of ETT for 15 days increased the risk of NI by 1.39 (CI 1.29,1.49) whilst the use of CVC and UC for 10 days increased the risk of NI by 1.73 (CI 1.52, 1.96) and 1.56 (CI 1.36,1.79) respectively.

Results of the multivariate analysis can be seen in Table 3. It shows the 3 variables adjusted for age, sex and postoperative condition that resulted associated to NI. The presence of ETT, time of use of ETT and time of use of CVC are associated to the development of NI.

**Table 3:** Multivariate analysis of children in the PICU

	RR	P > z	CI 95%	
***Uses ETT***				

No	Ref			

Yes	14.8	< 0.001	3.6	61.5

***Time of use of ETT***				

Days	1.01	0.024	1.00	1.02

***Time of use CVC***				

Days	1.03	0.005	1.01	1.05

RR: relative risk; CVC: central venous catheter; ETT: endotracheal tube

### Table 3: **Multivariate analysis of children in the PICU**

**Figure 4:**
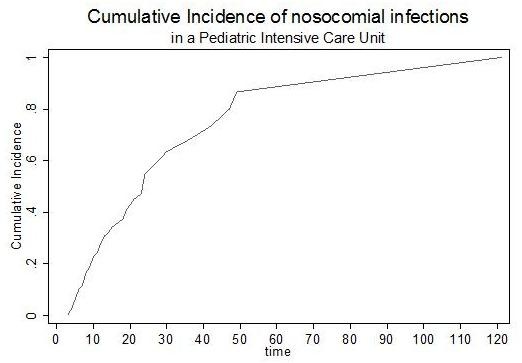
**Cumulative incidence of nosocomial infections in the Pediatric Intensive Care Unit**. Graphic shows the cumulative incidence (in days) of nosocomial infections.

The cumulative incidence of NI is shown in Figure 4.

### Figure 4. Cumulative incidence of nosocomial infections in the Pediatric Intensive Care Unit

## Discussion

Surveillance of NIs may help in decreasing the incidence of infections and reducing costs. In this study, 20% of patients in a PICU of a developing country developed an NI, BSI being the most frequent. Unexpectedly, *Candida *spp was the most common cause of BSI.

There are some limitations of this study, particularly when it is to be compared with others [1-4,6,13]. First, we studied only 3 types of NI. Secondly, our study did not aim to identify all nosocomial pneumonias but VAPs. Despite that 95% of nosocomial pneumonias are VAPs [20], this must be borne in mind when comparing figures. Finally, we did not search for viral agents, and it is known that virus can cause almost 20% of NIs in children [21].

NIs can be defined in different ways, thus assessment and comparison can be complicated. However, NIs are better expressed using as denominator the number of patients or number of days of use of devices. Use of devices is an indicator of patient's severity of illness or of invasive routines in ICU [22] and is strongly associated to development of BSI, pneumonia and UTI [3].

As in most pediatric studies [2-4], we found BSI as the most frequent NI. Only one study in Brazil [13] and another in Europe [1] found pneumonia to be the most common.

Our ratio for use of CVC, ETT and UC (0.46, 0.74 and 0.25 respectively) was, compared with NNIS reports (0.46, 0.43 and 0.32) [22], noticeably greater for ETT and similar regarding the use of CVC and UC. Despite similar use of CVC we had more BSI (18.1 vs. 7.3 - 8.9 per 1000 days of use of CVC [3,22]); this fact has also been described for NI in adult ICUs in developing countries [15]. It is likely that this increase in frequency of BSI is due to a greater susceptibility of our patients, differences in catheter insertion techniques or further care, or both. The aforementioned study [15] attributed the greater frequency of NI to less frequency of hand hygiene, low nurse-to-patient-ratios, and outdated technology.

The density of incidence for VAP and UTI seem to correspond to greater and similar use of ETT and UC, respectively. On the other hand, two studies regarding risk factors found 13.8 BSI/1000 days of use of CVC [23] and 11.6 VAP/1000 days of use of ETT [9], whilst a surveillance study for VAP found 8.9 VAP/1000 days of use of ETT [12]; these figures are closer to ours. Increased VAP rates could be due to greater rate of device use [22], as mentioned previously, but also to other factors. In our study, 30% of patients had a genetic syndrome, and the use of antibiotics (90%), sedation (75%), transfusions (70%), H_2 _blockers (60%), steroids (50%) and inotropes (40%) was highly prevalent; all these have been considered as potential risk factors for VAP [9,12].

The isolation of *Candida *spp. as the most common isolate in our children with BSI, in contrast with studies that show CNS as the principal causing agent [2-5,23,24], has already been pointed out [4]. There has been an increase in *Candida *BSI infections over the last years, as well as a shift toward non-*albicans Candida *[24,25]. Risk factors for candidemia are previous colonization, long ICU stays, presence of CVC, parenteral nutrition, MV, illness severity and prolonged use of antibiotics [25-27]. It is likely that the most important cause of our high prevalence of *Candida *is the extensive use of broad spectrum antibiotics[26]. Although at INSN vancomycin, carbapenems and ciprofloxacin must be approved by an infectologist before its use, third generation cephalosporins, vancomycin, and amikacin are frequently used at our institution, and more than 90% of PICU patients received any of these antibiotics or ciprofloxacin both in the PICU and previous to PICU admission. Long ICU stay is also described as a risk factor for candidemia [26], and although not described in the results section, children with BSI due to *Candida *had ICU stay almost twice longer than children with BSI due to other microorganisms (average, 53 vs. 27 days). Also not shown, previous surgery in children under 12 months was more frequently seen in children with BSI due to *Candida *than in children with BSI due to other microorganisms (75% vs. 29%). We have no explanation for this finding, but maybe it could be researched in future investigations. Other possible causes are previous colonization with *Candida*, due to the high use of antibiotics, as well as our high use of intubation and MV [26]. Candidemia is particularly important, because it is associated with high mortality, both in adults [28] and children, especially at lower ages and in those with comorbidities [26]. Due to its increasing numbers, more studies about risk factors for *Candida *infection are needed.

Gram negatives, mainly *Pseudomonas*, caused 80% of VAP in our study. *P aeruginosa *is the most common organism in children with VAP, followed by other Gram negatives and *S. aureus *[1,3,4,9,12,29,30]. There was just one case of *S. aureus *in our study, possibly because many children are admitted to the ICU having been treated with vancomicin.

The most common organisms in UTI are *E. coli *[2,3] or, as in our study, *Candida *[4,31]. We found 5 *Candida*, 4 of them were associated to urinary catheters.

We had a high rate of antibiotic resistance. All CNS causing BSI were resistant to oxacillin and around 80% of *Pseudomonas *in children with VAP were resistant to ceftazidime, amikacin, ciprofloxacin and meropenem. Resistance pattern is high at INSN: 50% of *S aureus *are resistant to oxacillin, 85% of *Klebsiella *are resistant to third generation cephalosporin and 50 - 90% of *P aeruginosa *are resistant to carbapenems, amikacin, ceftazidime and ciprofloxacin (source: Committee on Nosocomial Infections). We believe that this is due to the extensive use of antibiotics.

Mortality of NI in the present study was 38%, notably greater than that reported from PICUs of developed countries (7.7 - 10%) [1,2], and somewhat greater than that from a PICU of Brazil (28.3%) [13]. Mortality in Latin American countries tends to be greater than that in the USA. A study in 6 PICUs from this region found greater mortality than expected among lower risk patients (PRISM < 10), compared to one PICU in USA, which could be explained on the basis of increased use of central lines and ETT as well as broad spectrum antibiotics [32].

Described mortality for BSI is 14 - 26.5% [5,23,24]; we found 31%, this could be related to greater severity of disease, differences in management or both. Notwithstanding we found greater mortality (46%) for BSI due to *Candida *than with other organisms, the limited number does not allow us comparative analysis.

Nosocomial pneumonia is the main cause of death in children with NI [30], but it is doubtful if VAP increases mortality in children on MV. In contrast to other studies [9,12], our mortality for VAP was significantly greater than for children without VAP. In a study from a developing country, the risk adjusted PICU mortality was significantly greater in VAP versus non-VAP patients [33]. Mortality for VAP seems also be related to the causative pathogen and the time of onset [8,19]. For example, Gram negative bacilli (particularly *Pseudomonas*) have > 80% mortality [8], and early VAP has smaller mortality than late VAP [19]. Our limited numbers (5 early and 22 late VAPs) forbid further analysis, but we found a tendency to greater mortality in late VAP. We have found no pediatric studies that have researched on this topic beyond the neonatal area.

A striking feature of our data is that ICU stay previous to NI diagnosis was greater for this group than for children who did not develop an infection. It is known that NIs increase hospital stay [1,9,12,23], but also a longer stay *per se *is a risk factor for NI [34]. That suggests that patients who developed an NI were already significantly different from those who did not, which is particularly important for future studies and when interpreting the impact of the NI. Since a significant portion of our patients had co-morbidity, it is possible that its condition resulted in a longer stay, predisposing them to NI. By the other hand, although in bivariate analysis ICU stay was associated to NI development, in multivariate analysis it did not result significant.

This study has several restrictions, like the lack of inclusion of data on re-intubation, which could be relevant for VAP; likewise, the sample collecting technique (tracheal aspiration) is not the one recommended by most authors. PRISM score was recorded in only 22% of patients. Neither did we stratify cases according to severity of disease, which could influence the appearance of NI, mortality and stay. Finally, it must be borne in mind that the INSN is a national reference center and admits children from other institutions, thus our results may differ from other PICUs. Additionally, we were not able to calculate attributable mortality because data that could have influenced the results was not available.

## Conclusions

This study highlights the importance of NIs in children admitted to a PICU in a developing country. Patients in these units, despite representing a small percentage of inpatients, contribute with over 20% of NIs [35]. Potential impact of NI control is such that of 6 recommendations to improve interventions in health, issued by the Institute for Health Improvement, 3 are directly related to actions to decrease NIs [20]. In the ICU at the INSN one of every 5 children acquires an NI, which was associated with increased mortality and length of stay.

## Competing interests

The authors declare that they have no competing interests.

## Authors' contributions

**MRB **made substantial contributions to acquisition, analysis and interpretation of data, was involved in drafting the manuscript and approved its final version. **JAT **conceived the study, participated in design, analysis and interpretation of data, was involved in drafting the manuscript and approved its final version. **VJS **made substantial contributions to design, analysis and interpretation of data, was involved in drafting the manuscript and approved its final version. **MCA **made substantial contributions to acquisition and interpretation of data and approved its final version. **JLC **made substantial contributions to analysis and interpretation of data, was involved in drafting the manuscript and approved its final version. **FCU **made substantial contributions to acquisition and interpretation of data and approved its final version

## Pre-publication history

The pre-publication history for this paper can be accessed here:

http://www.biomedcentral.com/1471-2431/10/66/prepub
